# Combined ^15^N-Labeling and TandemMOAC Quantifies Phosphorylation of MAP Kinase Substrates Downstream of MKK7 in *Arabidopsis*

**DOI:** 10.3389/fpls.2017.02050

**Published:** 2017-12-08

**Authors:** Nicola V. Huck, Franz Leissing, Petra Majovsky, Matthias Buntru, Christina Aretz, Mirkko Flecken, Jörg P. J. Müller, Simon Vogel, Stefan Schillberg, Wolfgang Hoehenwarter, Uwe Conrath, Gerold J. M. Beckers

**Affiliations:** ^1^Department of Plant Physiology, Aachen Biology and Biotechnology, RWTH Aachen University, Aachen, Germany; ^2^Proteome Analytics, Leibniz Institute of Plant Biochemistry, Halle, Germany; ^3^Fraunhofer Institute for Molecular Biology and Applied Ecology IME, Aachen, Germany

**Keywords:** mitogen-activated protein kinases, *Arabidopsis thaliana*, plant immunity and development, MKK7 cascade, tandemMOAC

## Abstract

Reversible protein phosphorylation is a widespread posttranslational modification that plays a key role in eukaryotic signal transduction. Due to the dynamics of protein abundance, low stoichiometry and transient nature of protein phosphorylation, the detection and accurate quantification of substrate phosphorylation by protein kinases remains a challenge in phosphoproteome research. Here, we combine tandem metal-oxide affinity chromatography (tandemMOAC) with stable isotope ^15^N metabolic labeling for the measurement and accurate quantification of low abundant, transiently phosphorylated peptides by mass spectrometry. Since tandemMOAC is not biased toward the enrichment of acidophilic, basophilic, or proline-directed kinase substrates, the method is applicable to identify targets of all these three types of protein kinases. The MKK7-MPK3/6 module, for example, is involved in the regulation of plant development and plant basal and systemic immune responses, but little is known about downstream cascade components. Using our here described phosphoproteomics approach we identified several MPK substrates downstream of the MKK7-MPK3/6 phosphorylation cascade in *Arabidopsis*. The identification and validation of dynamin-related protein 2 as a novel phosphorylation substrate of the MKK7-MPK3/6 module establishes a novel link between MPK signaling and clathrin-mediated vesicle trafficking.

## Introduction

Mitogen-activated protein kinase (MPK) cascades are central signaling modules that transduce multiple extracellular stimuli into cellular responses (Arthur and Ley, 2013). Each MPK cascade is initiated by specific extracellular cues and leads to activation of one or more MPKs following the successive phosphorylation of a MPK kinase kinase (MEKK) and a MPK kinase (MKK or MEK) (MAPK Group et al., 2002; Rodriguez et al., 2010). In plants, MEKKs directly activate MKKs by dual phosphorylation of the phosphorylation site motif (S/T-X_5_-S/T) between kinase subdomains VII and VIII. MKKs, in turn, activate MPKs by dual phosphorylation of a conserved tripeptide (T–X–Y) motif located in the activation loop of the enzyme (MAPK Group et al., 2002). Once activated, MPKs phosphorylate diverse substrates that are mainly located in the cytosol and nucleus, to bring about changes in protein function and gene expression that ultimately result in the appropriate cellular response (Rodriguez et al., 2010; Meng and Zhang, 2013).

In plants, unambiguous identification and direct measurement of *in vivo* phosphorylated MPK substrates by mass spectrometry (MS) proved to be difficult mainly because of low stoichiometry and transient nature of these phosphorylation events (Zhang et al., 2016). Recently, we described a robust phosphoproteomics approach for the identification and label-free quantification of site-specific phosphorylation of low abundant protein substrates for MPKs in *Arabidopsis thaliana* (Hoehenwarter et al., 2013). The method, which we named tandemMOAC, represents a two-step chromatography combining phosphoprotein enrichment using Al(OH)_3_-based metal oxide affinity chromatography (MOAC), tryptic digest of enriched phosphoproteins, and TiO_2_-based MOAC to enrich phosphopeptides from complex protein samples. We applied tandemMOAC to transgenic conditional gain-of-function *Arabidopsis* plants that express the constitutively active *Nicotiana tabacum* MEK2^DD^ modified protein under control of the dexamethasone (DEX)-inducible *GVG* promoter. Due to the substitution of the conserved amino acids serine (S) and threonine (T) within the activation loop of *Nt*MEK2 by aspartic acid (D), the kinase is constitutively active (Ren et al., 2002; Liu and Zhang, 2004). Upon DEX treatment of transgenic *GVG::NtMEK2^DD^ Arabidopsis* plants, *Nt*MEK2^DD^ accumulates and specifically activates endogenous MPK3 and MPK6 that subsequently phosphorylate their *in vivo* targets. The successive enrichment of phosphoproteins in a first and phosphopeptide extraction in a second step strongly enriched the phosphoproteome. Our previous work demonstrated that subsequent label-free LC-MS/MS analyses allowed direct identification and site-specific quantification of differential phosphorylation events induced by *in planta* activation of MPK3 and MPK6 (Hoehenwarter et al., 2013). Unfortunately, the low peptide ion counts in tandemMOAC-enriched samples compelled us to perform manual integration of the peptide ion signal peaks for relative quantification of selected phosphopeptides. Nevertheless, our tandemMOAC strategy disclosed the identity of numerous novel phosphorylation sites and *in vivo* targets of MPKs, particularly of MPK3 and MPK6 (Hoehenwarter et al., 2013; Leissing et al., 2016).

*Arabidopsis* MKK7 was shown to modulate a variety of cellular processes. Molecular genetic analyses previously demonstrated that MKK7 is required for plant immunity (Zhang et al., 2007) but inhibits auxin signaling (Dai et al., 2006). Recently, MPK3 and MPK6 were both revealed as the *in vivo* substrates for phosphorylation by MKK7 and it was shown that the MKK7-MPK6 and MKK7-MPK3 cascades perform distinct functions *in planta* (Jia et al., 2016). While it was demonstrated that the MKK7-MPK6 module mainly contributes to plant growth and development, MKK7-MPK3 was hypothesized to contribute to plant immunity (Jia et al., 2016). However, until now, the identity of downstream components of the MKK7-MPK3/6 phosphorylation cascade remained elusive. In this study, we used a transgenic conditional overexpression system combined with an improved tandemMOAC protocol, including stable isotope labeling of whole *Arabidopsis* plants, for more accurate quantification of phosphopeptides. This approach allowed us to tackle the identification of substrates downstream of the *Arabidopsis* MKK7-MPK3/6 module.

## Materials and Methods

### Plant Growth and Treatment

Seedlings of *Arabidopsis thaliana pER8::cMYC-MKK7* Col-0 were grown in 50 mL MES-buffered, half-strength Murashige and Skoog medium with 4.70 mM potassium nitrate and 5.15 mM ammonium nitrate. Nitrogen salts were supplied either in normal ^14^N form or in ^15^N-enriched form (>98% ^15^N atom % K^15^NO_3_, ^15^NH_4_^15^NO_3_). Medium was supplemented with 2.5 g/L sucrose. Plants were grown at 22°C in continuous light (70 μE/m^2^/sec). Twelve-day-old seedlings were treated with β-estradiol (1 μM) in ethanol or with ethanol as the control and collected 6 h after treatment.

### Protein Extraction and MOAC-Enrichment of Phosphoproteins

Prior to protein extraction and fractionation, ground tissue powder of ^14^N and ^15^N-labeled was mixed in a 1:1 ratio. Phenolic total protein extraction and enrichment of phosphorylated proteins was performed as described before (Thomas et al., 2015). In brief, ground tissue powder was washed twice with ice-cold acetone before resuspension of the tissue pellet in 10% (w/v) trichloroacetic acid (TCA) in acetone and sonication for 10 min in a sonication bath. Pellets were washed twice with 10% (w/v) TCA in acetone, twice in 10% (w/v) TCA, and twice in 80% (v/v) acetone. Air-dried pellets were resuspended in dense SDS buffer (30% (w/v) sucrose, 2% (w/v) SDS, 0.1 M Tris-HCl pH 8.0, 5% (v/v) 2-mercaptoethanol) before adding Tris-buffered phenol and vigorous vortexing. The upper phenol phase was separated by centrifugation (15 min at 4,000 × *g*) and proteins were recovered by precipitation at -20°C (60 min) with 5 volumes of 0.1 M ammonium acetate in methanol. After centrifugation the protein pellets were washed twice with 0.1 M ammonium acetate in methanol and twice with 80% (v/v) acetone.

For Al(OH)_3_-based MOAC enrichment of phosphoproteins, protein pellets were re-solubilized in buffer A (30 mM MES pH 6.1, 8 M urea, 150 mM sodium L-glutamic acid, potassium L-aspartic acid, 20 mM imidazole, 0.25% (w/v) CHAPS). Phosphoprotein binding to Al(OH)_3_ (prewashed twice with buffer A) was achieved by incubation on a rotator at 10°C for 60 min. Al(OH)_3_-bound phosphoproteins were washed 6 times with buffer B (similar to buffer A, except that the concentration of amino acids was increased to 200 mM). Phosphoproteins were eluted in buffer C (200 mM potassium pyrophosphate pH 9.0, 8 M urea) by head-over-head incubation for 30 min at room temperature. After centrifugation (3,000 × *g* for 5 min) the phosphoprotein containing supernatant was loaded onto a Amicon Ultra-15 centrifugal filter unit (Merck, Darmstadt, Germany) to concentrate the phosphoprotein sample. The retentate was diluted with 4 volumes of ddH_2_O before addition of 0.01 volume of 2% (w/v) sodium deoxycholate. After vortexing and incubation for 5 min, 0.1 volume of 100% (w/v) TCA was added to precipitate phosphoproteins for 120 min on ice. Precipitated phosphoproteins were collected by centrifugation (14,000 × *g* for 10 min at 4°C) and subsequently washed once with 25% TCA in ddH_2_O, once with 80% acetone in 50 mM Tris-HCl pH 7.5, and once with 100% acetone, before air-dried pellets were stored at -20°C.

### Western Blotting and Immunodetection

Protein samples were subjected to SDS-PAGE, transferred to nitrocellulose, and used for immunodetection as described (Beckers et al., 2009). Strep-Tactin^®^ HRP conjugate was purchased from IBA Lifesciences (Göttingen, Germany); primary rabbit antibodies against the thiophosphatester (α-TPE) from Abcam (Cambridge, United Kingdom) all other antibodies were purchased from Cell Signaling Technology (Danvers, MA, United States). Chemiluminescence detection of antigen-antibody complexes was done with Immobilon^TM^ Western substrate (Merck, Darmstadt, Germany).

### Protein Digestion, Peptide Desalting, and MOAC-Enrichment of Phosphopeptides

Enrichment of phosphopeptides by MOAC was essentially done as described (Hoehenwarter et al., 2013). Briefly, 500 μg MOAC-enriched phosphoproteins were trypsin digested [Poroszyme immobilized trypsin (1/100 v/w) (Thermo Fisher Scientific, Waltham, MA, United States)] overnight before protein digests were desalted using a self-packed graphite (500 mg) spin column with a polyethylene filter of 10 μM pore size (MoBiTec, Göttingen, Germany). Meanwhile, graphite was equilibrated by washing twice with 500 μL of 1 M ammonia, once with 500 μL acetonitrile (ACN) and twice with 500 μL 1% (v/v) trifluoroacetic acid (TFA). Meanwhile phosphoprotein digestion mixture was acidified by adding 10% (v/v) TFA to bring the final concentration to 1.25% (v/v) TFA and centrifuged for 10 min at 16,000 × *g* before loading on the column. Graphite was washed twice with 500 μL 1% (v/v) TFA before peptides were eluted twice with 200 μL 0.1% (v/v) formic acid (FA) in 50% (v/v) ACN. Eluted peptides were pooled and dried in a vacuum concentrator. After dissolving peptides in 100 μL buffer A (phthalic acid-saturated 50% (v/v) ACN, 2.5% (v/v) TFA) they were loaded onto a TiO_2_ column (12.5 mg) pre-equilibrated with buffer A (25 mg TiO_2_ per 1 mg peptides). After phosphopeptide binding, TiO_2_ was washed twice with 250 μL buffer A, twice with 250 μL 50% (v/v) ACN, 0.1% (v/v) TFA, and twice with 250 μL 0.1% (v/v) TFA. Finally, phosphopeptides were eluted three times with 100 μL 5% (v/v) ammonia, eluates were pooled, dried in a vacuum concentrator and stored at -20°C until MS-analysis.

### Mass Spectrometry

Liquid chromatography and mass spectrometry (LC-MS) of phosphorylated peptides was described in detail (Thomas et al., 2015). In brief, peptides were separated using C18 reverse phase chemistry employing a pre-column (EASY column SC001, length 2 cm, inner diameter (ID) 100 μm, particle size 5 μm) in line with an EASY column SC200 with a length of 10 cm, ID of 75 μm and a particle size of 3 μm (both from Thermo Fisher Scientific). Peptides were eluted into a Nanospray Flex ion source (Thermo Fisher Scientific) with a 180 min gradient increasing from 5% to 35% (v/v) ACN in ddH_2_O and electrosprayed into an Orbitrap Velos Pro mass spectrometer (Thermo Fisher Scientific). The source voltage was set to 1.9 kV, the S Lens RF level to 50%. The delta multipole offset was –7.00.

Measurements employed a data dependent acquisition (DDA) scan strategy wherein up to 20 of the most abundant ions with a minimum signal of 1,000 recorded in an MS survey scan were isolated and subjected to collision induced dissociation (CID). The AGC target value was set to 1e06 and the maximum injection time (max IT) to 500 ms in the Orbitrap. The parameters were set to 1e04 and 100 ms in the LTQ with an isolation width of 2 Da for precursor isolation and MS/MS scanning. Multi stage activation (MSA) was applied to further dissociate fragment ion peaks resulting from neutral loss of the phosphate moiety by dissociation of the high-energy phosphate bond to generate b- and y-fragment ion series rich in peptide sequence information. Neutral loss masses were 24.49, 32.66, 49, 65.3, 98, 147, 195.94, and 293.91. The repeat count was set to 1 and the repeat duration to 30 s. The exclusion duration was set to 40 s and the exclusion width to 10 ppm.

### Identification and Quantification of Phosphorylated Peptides

Procedures were described in detail previously and performed with Mascot Distiller v.2.5.1.0 linked to an in-house Mascot server v.2.5.0 (Thomas et al., 2015). To determine the level of ^15^N incorporation into peptides, MS data was used to search the TAIR10 database with common contaminants amended (35,394 sequences and 14,486,974 residues) with the enzyme set to trypsin/P. The tolerated precursor and fragment ion mass errors were 7 ppm and 0.8 Da, respectively. Oxidation of methionine and phosphorylation of serine and threonine residues were set as variable modifications. The quantitation option was set to ^15^N metabolic. Peptides and proteins were identified and extracted ion currents (XIC) of naturally occurring and heavy isotope incorporated peptide pairs were fit to calculated ion currents of the respective peptide pairs with the corrections option iteratively set to impurity ^15^N 96%, 97%, 98%, 98.5%, and 99%. *R*^2^ values of all XIC to calculated ion current fits for all identified phosphopeptides for the set incorporation levels were extracted. The *R*^2^ distribution with the highest mode was used as an estimate of phosphopeptide ^15^N incorporation. The results of the search, XIC extraction and fitting obtained with the respective incorporation level was then used to identify and quantify phosphopeptides by calculating the XIC peak area ratio of the naturally occurring and ^15^N incorporated peptides of all peptide pairs (light to heavy ratio, L/H).

### Filtering and Statistical Analyses of Phosphoproteomics Data

Only the highest ranking phosphopeptide annotation for each MS/MS spectrum was kept and used for peptide quantification. Phosphopeptide L/H ratios that were more than 100-fold different from the median across the replicates were considered outliers and excluded from further analysis with the Perseus software platform version 1.5.5.3 (Tyanova et al., 2016). Phosphopeptide quantification ratios were log_2_-transformed and subsequently median-normalized before a one-sample *t*-test (*p* < 0.05) was performed on all phosphopeptides that were quantified in at least four sample replicates (Yang et al., 2013).

### *In Vitro* Transcription and Translation

StrepII-tagged eYFP, VQ4 (AT1G28280), HON5 (AT1G48620), DRP2B (AT1G59610), WRKY6 (AT1G62300), CLMP1 (AT1G62390), MKK2 (AT4G29810), and NOT2/3/5 (AT5G18230) were expressed in a coupled cell-free transcription–translation system based on tobacco BY-2 cell lysates as described (Buntru et al., 2015).

### Substrate Labeling Reactions

Immunocomplex kinase assays were essentially performed as described previously (Leissing et al., 2016). StrepII-tagged substrates were purified from tobacco BY-2 cell lysates using Strep-Tactin^®^ Macroprep^®^ affinity beads (IBA Lifesciences) according to manufacturer’s instructions.

## Results and Discussion

### ^15^N-Stable Isotope Labeling and TandemMOAC Extraction of Phosphopeptides

**FIGURE 1:**
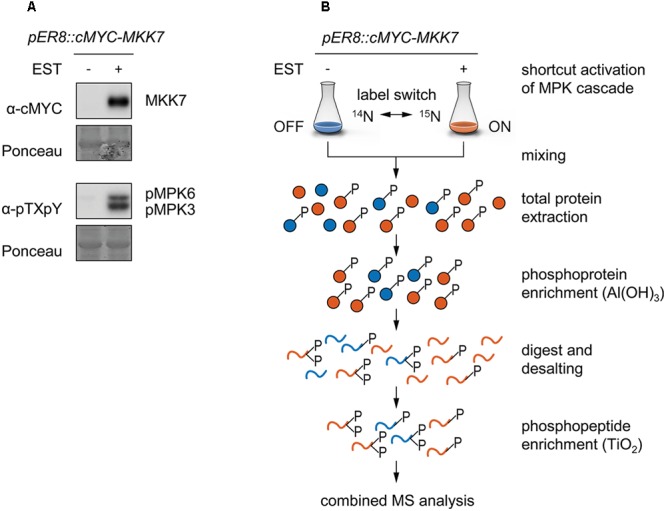
Metabolic labeling combined with tandemMOAC to identify phosphorylation substrates of MKK7-MPK3/6. Twelve-day old *pER8::cMYC-MKK7* seedlings were treated with ethanol as a control (-) or with 1 μM β-estradiol (+) to activate *cMYC-MKK7* transgene expression. Six hours later, seedlings were harvested and ground in liquid nitrogen to a fine powder. **(A)** Total protein was extracted and analyzed by SDS-PAGE, western blotting analysis, and immunodetection with antibodies to examine expression of the transgene (cMYC) and phosphorylation of MPK3/6 (pTXpY). The membrane was subjected to Ponceau S staining to check equal gel loading. **(B)** Reciprocal ^14^N- and ^15^N-labeled *Arabidopsis* seedlings were ground separately and subsequently mixed in a 1:1 (w/w) ratio. Total protein was extracted, phosphoproteins enriched via Al(OH)_3_-based MOAC and digested with trypsin before peptides were desalted using graphite columns. After TiO_2_-based MOAC enrichment of phosphopeptides, samples were analyzed by LC-MS/MS.

To illuminate the identity of downstream targets of *Arabidopsis* MKK7, we used transgenic *pER8::cMYC-MKK7* plants conditionally expressing a cMYC-tagged form of *At*MKK7 from the β-estradiol-inducible promoter (pER8) (Zuo et al., 2000). Treatment of these transgenic conditional overexpression plants with the steroid hormone ß-estradiol leads to accumulation of cMYC-MKK7 that specifically phosphorylates endogenous MPK3 and MPK6 (**Figure 1A**) (Jia et al., 2016). Next, we embarked on a reciprocal ^15^N-stable isotope metabolic labeling-based quantitative phosphoproteomic analysis to accurately quantify differentially regulated tandemMOAC-extracted phosphopeptides. Transgenic *pER8::cMYC-MKK7* seedlings were grown in medium containing either the natural abundance of nitrogen isotope (^14^N) salts, or stable “heavy” nitrogen isotope (^15^N, >98%) enriched salts, as the sole nitrogen source (**Figure 1B**). This metabolic labeling approach led to an ^15^N incorporation of nearly 98% as determined by quality of fit analysis of measured and calculated XICs taking into account different levels of possible ^15^N incorporation (Supplementary Figure S1). After 12 days, seedlings were treated with ß-estradiol (or ethanol as the solvent control) to induce the transgene and activate the MKK7-MPK3/6 module. At the 6-h time point post induction seedlings were harvested and ground in liquid nitrogen before ^14^N and ^15^N-labeled tissue powder was combined in a 1:1 ratio. In total, we processed eight combined ^14/15^N-labeled tandemMOAC-enriched phosphopeptide samples by LC-MS/MS; this set comprised four biological replicate experiments. Each replicate included two reciprocal ^14/15^N-labeled samples. One of which was inversely labeled with heavy nitrogen (label switch) (**Figure 1B**). In a first step, phosphoproteins were enriched from denatured total protein samples by Al(OH)_3_-based MOAC before enriched phosphoprotein fractions were digested with trypsin. In a second step, the resulting peptide mixture was desalted via reverse-phase chromatography using graphite columns before phosphopeptides were enriched by TiO_2_-MOAC. Subsequent LC-MS/MS analysis of all tandemMOAC samples together yielded 9,324 MS/MS spectra annotated as phosphopeptides (pPSMs) using Mascot, resulting in the identification of 1,650 nonredundant phosphopeptide sequences with high confidence scores (Mascot identity threshold <0.05). These phosphopeptides mapped to 764 identified phosphoproteins.

### Quantitative Phosphopeptide Profiling of Inducible MKK7 Overexpression Plants

**FIGURE 2:**
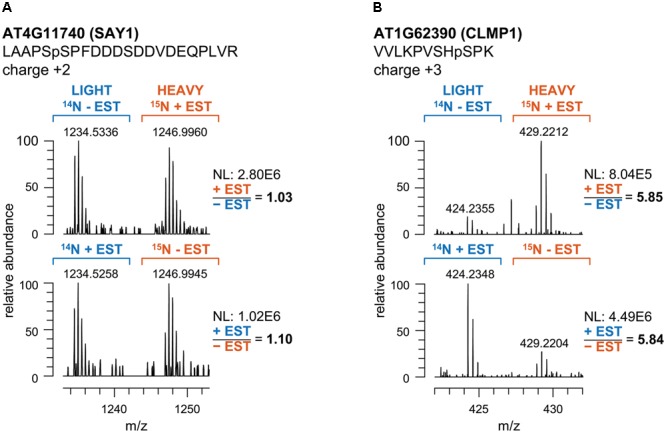
Quantitative analysis of the intensity ratios of labeled and unlabeled peptide pairs. **(A)** Mass spectra of the phosphopeptide LAAPSpSPFDDDSDDVDEQPLVR mapping to AT4G11740 (SAY1) in tandemMOAC extracted samples of reciprocally^14^N- and ^15^N-labeled seedlings treated with ethanol as a control (-EST) or with β-estradiol (+EST). m/z represents the ratio of ion mass over the charge of each phosphopeptide ion. **(B)** Same as in **(A)** but spectra correspond to the VVLKPVSHpSPK phosphopeptide of AT1G62390 (CLMP1). NL, normalized intensity level (counts per second).

**FIGURE 3:**
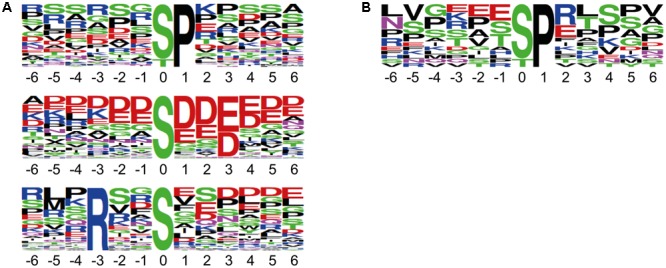
Sequence motifs of phosphorylation sites identified by tandemMOAC. Sequence logos of the phosphorylation sites mapped to peptide amino acid sequences generated by Motif-X with a significance of 0.000001 and the IPI *Arabidopsis* proteome as background. **(A)** Phosphorylation motifs enriched from all phosphopeptides identified in the presented dataset. **(B)** The proline-directed phosphorylation motif overrepresented among the significantly regulated peptides induced upon *in planta* expression of MKK7.

We performed reciprocal labeling within each biological quadruplicate experiment to allow treatment effects, such as labeling effects, biological variation or quantitation errors to be distinguished from experimental bias (Schulze and Usadel, 2010). In each sample, light and heavy labeled forms of a specific phosphopeptide were recorded as distinct, mass shifted XIC peaks. The ratio of peak areas of the light and heavy forms of each peptide was therefore calculated based on the XICs of monoisotopic peaks and the isotopologue distribution. Peptide quantification for exemplarily forward and reciprocal labeled samples is shown for two representative phosphopeptides in **Figure 2**. Mascot analysis of all tandemMOAC samples together yielded peptide abundance ratios for all 1,650 unique phosphopeptides. To confidently quantify phosphopeptides we only evaluated phosphopeptides recorded in at least four of the replicates. From this set of 454 phosphopeptides we extracted overrepresented phosphorylation patterns using Motif-X (**Figure 3A**) (Schwartz and Gygi, 2005; Chou and Schwartz, 2011). We discovered consensus phosphorylation motifs that resemble three main categories of S/T kinase recognition sequences: acidophilic-, basophilic-, and proline-directed phosphorylation sites (Douglass et al., 2012). This result indicates that tandemMOAC-enrichment of phosphopeptides is not biased toward enrichment of phosphorylated peptides that belong to either of these categories.

### Identification of MKK7 Downstream Targets

**FIGURE 4:**
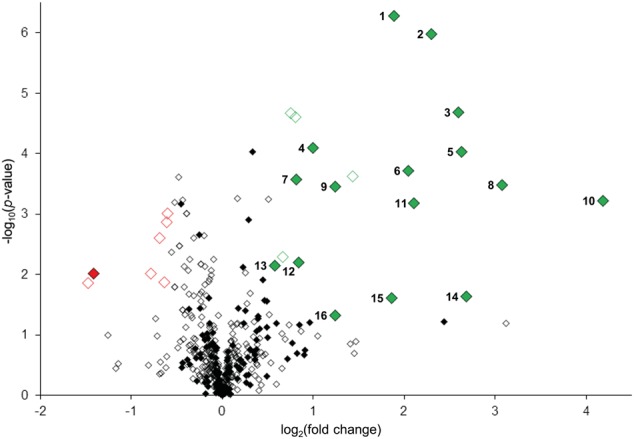
Quantitative analyses of MKK7-MPK3/6 induced changes in protein phosphorylation. Volcano plot representation of quantitative phosphoproteomic analysis of ethanol (control) and β-estradiol-treated transgenic *pER8::cMYC-MKK7* seedlings. Diamonds represent phosphopeptides quantified in ≥4 sample replicates, each phosphopeptide log_2_(fold change) is the average logarithmic ratio of phosphopeptide abundance of estradiol-treated versus control-treated seedlings plotted against the –log_10_(*p*-value) determined using Student’s *t*-test. Significantly down- and upregulated (*p* < 0.05, ≥1.5-fold change) phosphopeptides are, respectively, represented in red or green diamonds. Closed diamonds represent phosphopeptides specifically phosphorylated at serine or threonine directly followed by proline.

To confidently identify phosphorylation targets of the MKK7-MPK3/6 cascade, a one-sample Student’s *t*-test with *p* < 0.05 was performed with the above mentioned 454 phosphopeptides that were quantified in at least half of the sample replicates. Statistical analyses revealed that in total 27 phosphorylated peptides differed in their abundance at least 1.5-fold upon *in planta* induction of MKK7 (Supplementary Table S1). Graphical representation of these quantitative phosphoproteomics data in a volcano plot revealed that the abundance of 20 phosphopeptides was significantly enhanced ≥1.5-fold, whereas the abundance of only seven phosphopeptides was reduced (**Figure 4**). Within this set of 27 regulated phosphopeptides the Motif-X algorithm discovered a single predominant consensus phosphorylation motif reminiscent of the minimal motif required for MPKs (**Figure 3B**) (Alvarez et al., 1991). The latter suggests that most phosphoproteins that differ in their abundance upon ß-estradiol-induced accumulation of MKK7 are targeted by its downstream proline-directed MPKs. Accordingly, a phosphorylated MPK substrate motif was found in the sequence of 16 of the 20 accumulating phosphopeptides (**Table 1**). In contrast, the abundance of only a single phosphorylated MPK-targeted phosphopeptide was reduced. Together, these results indicate that ß-estradiol treatment of transgenic *pER8::cMYC-MKK7* seedlings specifically induces the phosphorylation of downstream targets of the MKK7-MPK3/6 cascade. The MPK motif-containing peptides that are phosphorylated upon activation of the MKK7-MPK3/6 cascade map to 14 full-length phosphoproteins (**Table 1**). Except the CCCH-type zinc finger protein 56 (AT5G12850) all substrates contain a kinase-interaction motif characterized by a cluster of basic residues positioned amino-terminal to a (L/I)X(L/I) motif (Supplementary Table S2) (Barsyte-Lovejoy et al., 2002). This corroborates our assertion that these proteins are likely direct *in vivo* substrates of MPK3/6. Although mostly novel, some of the proteins have been reported MPK3/6 substrates (Hoehenwarter et al., 2013; Pecher et al., 2014). When examining this set of direct phosphorylation targets of MKK7-MPK3/6 we found that many of them are DNA/RNA-binding proteins that predominantly localize to the nucleus (Supplementary Figures S2A,B). Furthermore, the enrichment of proteins in the GO biological process category ‘response to biotic and abiotic stimuli’ supports the notion that MKK7 is involved in stress-responsive signal transduction pathways in *Arabidopsis* (Supplementary Figure S2C).

**Table 1:** Identified and quantified *in vivo* substrate candidate proteins of the MKK7-MPK3/6 module.

ATG	Protein description	Peptide sequence										
		Ratio EST +/- (sample 1–8)								Score	m z^-1^	*z*
		1	2	3	4	5	6	7	8		Mean ratio	*p*-value
AT1G62390	CLUMPED CHLOROPLASTS 1 (CLMP1)	(1) VVLKPVSHpSPK								44	424.2	3
		4.1	3.6	5.1	3.3	3.0	4.9	3.2	2.9		**3.8**	**5.25E-07**
AT1G28280	VQ MOTIF-CONTAINING PROTEIN (VQ4)	(2) LLPLFPVTpSPR								46	660.4	2
		5.9		6.8	3.5	5.4	4.6	4.4	4.6		**5.0**	**1.05E-06**
AT1G62300	WRKY6	(3) LGREEpSPETESNKIQK								39	642.3	3
		6.7	7.6	6.6	17.0	3.8	3.5	4.2	5.5		**6.9**	**2.09E-05**
		(8) LGREEpSPETESNK								52	787.3	2
		12.0	4.7		12.6		9.2		6.6		**9.0**	**3.27E-04**
AT1G10290	DYNAMIN-RELATED PROTEIN 2A (DRP2A)	(4) AAAASSYSDNSGTESpSPR								92	919.4	2
		2.4	1.6	2.1	1.9			1.8	2.2		**2.0**	**8.05E-05**
AT4G38710	GLYCINE-RICH PROTEIN	(5) TLPVAVVEVVKPEpSPVLVIVEKPK								87	892.8	3
		12.3	4.2	5.6	3.4	3.6	20.5	4.0	7.4		**7.6**	**9.34E-05**
AT1G07110	”FRUCTOSE-2.6-BISPHOSPHATASE” (F2KP)	(6) SVETLpSPFQQK								45	672.3	2
		3.7		3.9	4.9		4.1				**4.2**	**1.93E-04**
AT4G29810	MAP KINASE KINASE 2 (MKK2)	(7) IISQLEPEVLpSPIKPADDQLSLSDLDMVK								59	1,091.9	3
		1.9	1.8	2.6	1.5	1.3	2.3	1.7	1.4		**1.8**	**2.71E-04**
AT1G59610	DYNAMIN-RELATED PROTEIN 2B (DRP2B)	(9) AAAASSWSDNSGTESpSPR								86	930.9	2
		2.6	2.4	2.1	2.4						**2.4**	**3.49E-04**
AT5G18230	TRANSCRIPTION REGULATOR NOT2/NOT3/NOT5 FAMILY PROTEIN	(10) NIMGVESNVQPLTpSPLSK								88	997.5	2
			7.9		25.4		11.0	41.2	21.7		**21.5**	**6.08E-04**
AT4G38550	*Arabidopsis* PHOSPHOLIPASE (pEARLI4)-LIKE PROTEIN	(11) NSSPPpSPFHPAAYK								66	790.4	2
			4.6	8.2	3.0	5.8	4.7		2.1		**4.7**	**6.66E-04**
		(16) STPGSPAHPPGARpSPPPSYLSNK								42	794.7	3
		4.1	1.7	3.9		3.3	0.8				**2.8**	**4.71E-02**
AT1G48620	HIGH MOBILITY GROUP A5 (HON5)	(12) KDGTpSPTVKPAASVSGGVETVK								37	740.7	3
		2.0	1.8	2.1	1.4						**1.8**	**6.30E-03**
AT4G15545	UNCHARACTERIZED PROTEIN	(13) HSSIQSQQASEAIEPAATDNENDAPKPSLSASLPLVSQTTpTPR								50	1,139.3	4
				1.3		1.7	1.2	1.9	1.4		**1.5**	**7.19E-03**
AT1G27100	ACTIN CROSS-LINKING PROTEIN	(14) RPTSSPLSAEpSPR								50	732.8	2
			7.4		10.4		12.2		1.8		**8.0**	**2.30E-02**
AT5G12850	ZINC FINGER CCCH DOMAIN-CONTAINING PROTEIN 56	(15) TLNPSNLEELFSAEVApSPR								92	1,077.5	2
		3.1		2.4	5.6	1.3	11.6				**4.8**	**2.49E-02**

*In addition to the calculated mean ratio, for each peptide β-estradiol-to-control ratios (EST +/–) are provided separately for each of the eight samples. Capital letters in the peptide sequence column indicate amino acids and lower case p indicates phosphorylation of the following S/T-P phosphorylation motif. Mean ratios and corresponding *p*-values are highlighted in bold. EST, β-estradiol; m z^-*1*^, mass over charge.*

### Multiple MKK7 Targets Are Directly Phosphorylated by MPK3 and 6

**FIGURE 5:**
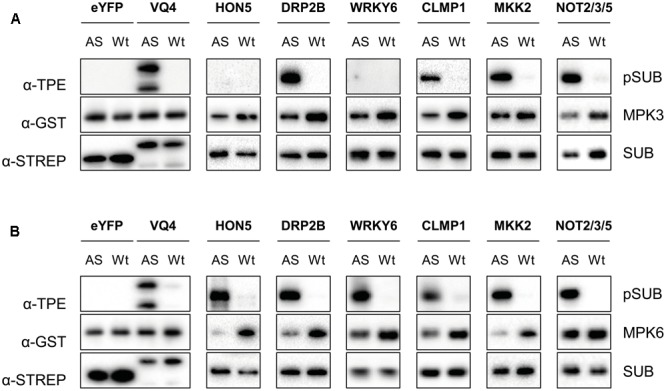
*In vitro* thiophosphorylation assay of MPK3/6. **(A)** Thiophosphorylation assays of GST-tagged wild type (Wt)- and analog-sensitive (AS)-MPK3 in the presence of Bn-ATPγS as cofactor. Tested StrepII-tagged substrates (SUB, e.g., eYFP, VQ4, HON5, DRP2B, WRKY6, CLMP1, MKK2, and NOT2/3/5) were purified from cell-free *in vitro* transcription-translation reactions using tobacco BY-2 cell lysates. Western blotting analysis, and immunodetection with specific antibodies was performed to check equal amounts of kinase (α-GST) and substrate (α-STREP) in each reaction and to examine phosphorylation of substrates (α-TPE). **(B)** Same as in **(A)** but using Wt- and AS-MPK6.

Recently, it has been shown that MKK7 phosphorylates MPK3 and MPK6 in response to pathogen infection and/or during plant growth and development (Jia et al., 2016). To confirm the phosphorylation of targets of MKK7 listed in **Table 1**, we tested the ability of MPK3/6 to directly phosphorylate a selected set of these proteins. In total, six strepII-tagged proteins (HON5, DRP2B, WRKY6, CLMP1, MKK2, and NOT2/3/5) were purified with Strep-Tactin^®^ Macroprep resin after successful cell-free protein synthesis in tobacco BY2 cell lysates (Supplementary Figure S3) (Buntru et al., 2015). Subsequent phosphorylation assays were performed with pre-activated, wild-type, and engineered analog-sensitive AS-MPK3 and AS-MPK6 in the presence of the bulky ATP analog N^6^-benzyl-ATPγS (Bn-ATPγS) as a co-factor (**Figure 5** and Supplementary Figure S4) (Leissing et al., 2016). Mutation of the so-called gatekeeper amino acid which enlarges the ATP-binding pocket of the kinase allows the AS kinase to catalyze thiophosphorylation of its substrate proteins. VQ4 served as a positive control for MPK3/6, as it was shown by us and others that this VQ-motif containing protein is specifically phosphorylated by MPK3/6 *in vivo* (Hoehenwarter et al., 2013; Pecher et al., 2014). In addition, *in vitro* synthesized eYFP served as a negative control protein in the kinase assays. All the phosphorylation targets of the MKK7-MPK3/6 module we tested were directly phosphorylated by AS-MPK6, whereas AS-MPK3 was unable to phosphorylate HON5 and WRKY6. Thus, most of the targets identified by the tandemMOAC approach are direct MPK3/6 substrates, at least *in vitro* (**Figure 5**).

Remarkably, in this study we found that upon *in planta* expression of MKK7 two functionally redundant Dynamin proteins of *Arabidopsis*, DRP2A, and DRP2B, are phosphorylated by MPK3/6 at a highly conserved serine residue (**Table 1** and **Figure 5**). Although their precise mode of action has yet to be disclosed, these proteins have been shown to play a key role in different membrane scission events (Taylor, 2011). For instance, DRP2 has been shown to localize to the Golgi apparatus where it enables trafficking of cargo molecules from the trans-Golgi network to the central vacuole (Jin et al., 2001; Huang et al., 2015). In addition, DRP2 colocalizes with clathrin light chain at the plasma membrane where dynamin proteins are thought to act in endocytosis by assembling as spirals around the neck of invaginating endocytic vesicles. Recently, DRP2B was also shown to play an important role in plant immunity (Smith et al., 2014). Mutant *drp2b* plants are impaired in ligand-induced endocytosis of the flagellin receptor FLS2 and are more susceptible to bacterial pathogens. Although the present study identified DRP2 as a downstream target of the MKK7-MPK3/6 phosphorylation module, the role of MPK-dependent phosphorylation of DRP2 in endocytosis remains unclear. Since the MKK7-MPK3/6 module is involved in basal and systemic plant immunity, it is tempting to speculate that the module-mediated DRP2 phosphorylation contributes to the overall plant immune response.

## Conclusion

In this work, we used a quantitative phosphoproteomic strategy based on ^15^N-labeling of *pER8::cMYC-MKK7* seedlings coupled to tandemMOAC enrichment of phosphoproteins and phosphopeptides to identify phosphorylation targets downstream of the MKK7-MPK3/6 module. We showed that the metabolic ^15^N-labeling/tandemMOAC approach represents a powerful novel tool that facilitates large-scale discovery and accurate relative quantification of low abundant phosphopeptides. We disclosed the identity of numerous novel phosphorylation sites and *in vivo* targets of MPKs, particularly MPK3 and MPK6. Because tandemMOAC is not biased toward the analysis of MPK substrate phosphorylation, the method will also be applicable to identify targets of members of other protein kinase families.

## Data Availability

The proteomics data have been deposited to the ProteomeXchange Consortium (Vizcaíno et al., 2014) (http://proteomecentral.proteomexchange.org) via the PRIDE partner repository with the dataset identifier PXD008200.

## Author Contributions

NH, FL, UC, and GB designed the research; NH, FL, PM, CA, MF, JM, and WH performed the experiments; MB, SV, and SS provided *in vitro* translated proteins; NH, FL, WH, and GB analyzed the data; UC and GB coordinated and helped to draft the manuscript.

## Conflict of Interest Statement

The authors declare that the research was conducted in the absence of any commercial or financial relationships that could be construed as a potential conflict of interest.
