# Supplementary material for: Combined 15N-Labeling and TandemMOAC Quantifies Phosphorylation of MAP Kinase Substrates Downstream of MKK7 in Arabidopsis
Source: Front Plant Sci. 2017 Dec 8;8:2050. doi: 10.3389/fpls.2017.02050 (PMC5727051; doi:10.3389/fpls.2017.02050)
Supplement: Supplementary file 1 [file Data_Sheet_1.DOCX]

SUPPLEMENTARY MATERIAL

**Table S1. All phosphopeptides significantly regulated upon *in planta* expression of MKK7 (p≤0.05).**

| **A) ATG** | **phosphopeptide sequence**  **(MPK substrate motif)** | **ratio EST +/- (sample 1-8)** | | | | | | | | **mean ratio**  **≥ 1,5** | **p-value**  **≤ 0,05** |
| --- | --- | --- | --- | --- | --- | --- | --- | --- | --- | --- | --- |
|  |  | **1** | **2** | **3** | **4** | **5** | **6** | **7** | **8** |  |  |
| **AT1G62390** | VVLKPVSHpSPK | 4,1 | 3,6 | 5,1 | 3,3 | 3,0 | 4,9 | 3,2 | 2,9 | **3,8** | **5,25E-07** |
| **AT1G28280** | LLPLFPVTpSPR | 5,9 |  | 6,8 | 3,5 | 5,4 | 4,6 | 4,4 | 4,6 | **5,0** | **1,05E-06** |
| **AT1G62300** | LGREEpSPETESNKIQK | 6,7 | 7,6 | 6,6 | 17,0 | 3,8 | 3,5 | 4,2 | 5,5 | **6,9** | **2,09E-05** |
| **AT1G10290** | AAAASSYSDNSGTESpSPR | 2,4 | 1,6 | 2,1 | 1,9 |  |  | 1,8 | 2,2 | **2,0** | **8,05E-05** |
| **AT4G38710** | TLPVAVVEVVKPEpSPVLVIVEKPK | 12,3 | 4,2 | 5,6 | 3,4 | 3,6 | 20,5 | 4,0 | 7,4 | **7,6** | **9,34E-05** |
| **AT1G07110** | SVETLpSPFQQK | 3,7 |  | 3,9 | 4,9 |  | 4,1 |  |  | **4,2** | **1,93E-04** |
| **AT4G29810** | IISQLEPEVLpSPIKPADDQLSLSDLDMVK | 1,9 | 1,8 | 2,6 | 1,5 | 1,3 | 2,3 | 1,7 | 1,4 | **1,8** | **2,71E-04** |
| **AT1G62300** | LGREEpSPETESNK | 12,0 | 4,7 |  | 12,6 |  | 9,2 |  | 6,6 | **9,0** | **3,27E-04** |
| **AT1G59610** | AAAASSWSDNSGTESpSPR | 2,6 | 2,4 | 2,1 | 2,4 |  |  |  |  | **2,4** | **3,49E-04** |
| **AT5G18230** | NIMGVESNVQPLTpSPLSK |  | 7,9 |  | 25,4 |  | 11,0 | 41,2 | 21,7 | **21,5** | **6,08E-04** |
| **AT4G38550** | NSSPPpSPFHPAAYK |  | 4,6 | 8,2 | 3,0 | 5,8 | 4,7 |  | 2,1 | **4,7** | **6,66E-04** |
| **AT1G48620** | KDGTpSPTVKPAASVSGGVETVK | 2,0 | 1,8 | 2,1 | 1,4 |  |  |  |  | **1,8** | **6,30E-03** |
| **AT4G15545** | HSSIQSQQASEAIEPAATDNENDAPKPSLSASLPLVSQTTpTPR |  |  | 1,3 |  | 1,7 | 1,2 | 1,9 | 1,4 | **1,5** | **7,19E-03** |
| **AT1G27100** | RPTSSPLSAEpSPR |  | 7,4 |  | 10,4 |  | 12,2 |  | 1,8 | **8,0** | **2,30E-02** |
| **AT5G12850** | TLNPSNLEELFSAEVApSPR | 3,1 |  | 2,4 | 5,6 | 1,3 | 11,6 |  |  | **4,8** | **2,49E-02** |
| **AT4G38550** | STPGSPAHPPGARpSPPPSYLSNK | 4,1 | 1,7 | 3,9 |  | 3,3 | 0,8 |  |  | **2,8** | **4,71E-02** |

| **B) ATG** | **phosphopeptide sequence**  **(MPK substrate motif)** | **ratio EST +/- (sample 1-8)** | | | | | | | | **mean ratio**  **≤ 0,67** | **p-value**  **≤0,05** |
| --- | --- | --- | --- | --- | --- | --- | --- | --- | --- | --- | --- |
|  |  | **1** | **2** | **3** | **4** | **5** | **6** | **7** | **8** |  |  |
| **AT4G05150** | ISpTPELPPPVFIKPEpSPEPVSTPK | 0,3 | 0,5 | 0,3 |  |  | 0,5 |  |  | **0,4** | **9,54E-03** |

| **C) ATG** | **phosphopeptide sequence** | **ratio EST +/- (sample 1-8)** | | | | | | | | **mean ratio**  **≥ 1,5** | **p-value**  **≤0,05** |
| --- | --- | --- | --- | --- | --- | --- | --- | --- | --- | --- | --- |
|  |  | **1** | **2** | **3** | **4** | **5** | **6** | **7** | **8** |  |  |
| **AT1G10140** | IEEEEETEEGpSVNPK |  | 2,7 | 4,2 | 2,7 | 2,9 |  | 2,2 | 2,0 | **2,8** | **2,40E-04** |
| **AT4G31700** | SRLpSSAAAKPSVTA | 2,0 | 1,9 | 2,0 | 1,7 | 1,8 | 1,4 | 1,9 | 1,3 | **1,8** | **2,49E-05** |
| **AT1G76070** | SIFpSFSPASGR |  | 1,6 | 1,8 |  | 1,9 | 1,5 | 1,7 | 1,7 | **1,7** | **2,15E-05** |
| **AT5G61210** | SSKPNPFDpSDDESDNKHTLNPSK | 1,9 | 1,4 |  | 1,5 |  |  | 1,3 | 2,0 | **1,6** | **5,14E-03** |

| **D) ATG** | **phosphopeptide sequence** | **ratio EST +/- (sample 1-8)** | | | | | | | | **mean ratio**  **≤ 0,67** | **p-value**  **≤0,05** |
| --- | --- | --- | --- | --- | --- | --- | --- | --- | --- | --- | --- |
|  |  | **1** | **2** | **3** | **4** | **5** | **6** | **7** | **8** |  |  |
| **AT2G38280** | SHpSVSGDLHGVQPDPIAADILR |  |  | 0,5 | 0,7 | 0,7 | 0,7 | 0,7 | 0,7 | **0,7** | **9,88E-04** |
| **AT4G15545** | RHSVpSFATTR |  |  | 0,6 | 0,7 |  | 0,6 |  | 0,6 | **0,7** | **1,34E-03** |
| **AT5G21940** | SFpSVADFPR | 0,7 |  | 0,5 |  | 0,6 | 0,8 |  |  | **0,7** | **1,35E-02** |
| **AT3G53460** | SGGYGpSERGGGYGSER |  | 0,6 | 0,5 | 0,7 |  | 0,7 |  |  | **0,6** | **2,48E-03** |
| **AT5G64850** | DASRApSLGNEEELINPFHDQPPVDTAKPK | 0,3 | 0,5 | 0,6 | 0,6 | 0,7 |  | 0,9 | 0,6 | **0,6** | **9,66E-03** |
| **AT5G28770** | RVEpSLEHLQK | 0,2 |  |  | 0,6 | 0,5 | 0,6 | 0,2 |  | **0,4** | **1,37E-02** |
|  |  |  |  |  |  |  |  |  |  |  |  |

**Table S2. Presence of kinase interaction motifs (KIM) within the primary sequence of *in vivo* MPK substrate candidates.** Putative KIMs are indicated in red, recorded phosphopeptide sequences are underlined, lower case p indicates phosphorylation of the following Ser or Thr residue.

| **ATG** | **protein sequence** |
| --- | --- |
| **AT1G62390** | MGKSGGRKKKSGGSNSNSSQVNSSETSGLSKPSTIVNGGVDFDASIFLKRAHELKEEGNKKFQARDYVGALEQYENGIKLIPKSHPDRAVFHSNRAACLMQMKPIDYESVISECSMALKSQPGFTRALLRRARAFEAVGKFDLAVQDVNVLLGSDPNHKDAGEISKRLKTALGPHQDLQSRPSPAALGASAALGGPIAGLGPCLPSRNVHKKGVTSPVGSVSLPNASNGKVERPQVVNPVTENGGSVSKGQASRVVLKPVSHpSPKGSKVEELGSSSVAVVGKVQEKRIRWRPLKFVYDHDIRLGQMPVNCRFKELREIVSSRFPSSKAVLIKYKDNDGDLVTITSTAELKLAESAADCILTKEPDTDKSDSVGMLRLHVVDVSPEQEPMLLEEEEEEVEEKPVIEEVISSPTESLSETEINTEKTDKEVEKEKASSSEDPETKELEMDDWLFDFAHLFRTHVGIDPDAHIDLHELGMELCSEALEETVTSEKAQPLFDKASAKFQEVAALAFFNWGNVHMCAARKRIPLDESAGKEVVAAQLQTAYEWVKERYTLAKEKYEQALSIKPDFYEGLLALGQQQFEMAKLHWSYLLAQKIDISGWDPSETLNLFDSAEAKMKDATEMWEKLEEQRMDDLKNPNSNKKEEVSKRRKKQGGDGNEEVSETITAEEAAEQATAMRSQIHLFWGNMLFERSQVECKIGKDGWNKNLDSAVERFKLAGASEADIATVVKNHCSNEAAATEGDEKKVPAP |
| **AT1G28280** | MENSPRYREATNLIPSPRCHNSNNSCGMSSSSESNKPPTTPTRHVTTRSESGNPYPTTFVQADTSSFKQVVQMLTGSAERPKHGSSLKPNPTHHQPDPRSTPSSFSIPPIKAVPNKKQSSSSASGFRLYERRNSMKNLKINPLNPVFNPVNSAFSPRKPEILSPSILDFPSLVLSPVTPLIPDPFDRSGSSNQSPNELAAEEKAMKERGFYLHPSPATTPMDPEPRLLPLFPVTpSPRVSGSSSASTS |
| **AT1G62300** | MDRGWSGLTLDSSSLDLLNPNRISHKNHRRFSNPLAMSRIDEEDDQKTRISTNGSEFRFPVSLSGIRDREDEDFSSGVAGDNDREVPGEVDFFSDKKSRVCREDDEGFRVKKEEQDDRTDVNTGLNLRTTGNTKSDESMIDDGESSEMEDKRAKNELVKLQDELKKMTMDNQKLRELLTQVSNSYTSLQMHLVSLMQQQQQQNNKVIEAAEKPEETIVPRQFIDLGPTRAVGEAEDVSNSSSEDRTRSGGSSAAERRSNGKR(LGREEpSPETESNK)IQKVNSTTPTTFDQTAEATMRKARVSVRARSEAPMISDGCQWRKYGQKMAKGNPCPRAYYRCTMATGCPVRKQVQRCAEDRSILITTYEGNHNHPLPPAAVAMASTTTAAANMLLSGSMSSHDGMMNPTNLLARAVLPCSTSMATISASAPFPTVTLDLTHSPPPPNGSNPSSSAATNNNHNSLMQRPQQQQQQMTNLPPGMLPHVIGQALYNQSKFSGLQFSGGSPSTAAFSQSHAVADTITALTADPNFTAALAAVISSMINGTNHHDGEGNNKNQ |
| **AT1G10290** | MEAIDELSQLSDSMKQAASLLADEDPDETSSSKRPATFLNVVALGNVGAGKSAVLNSLIGHPVLPTGENGATRAPIIIELSRESSLSSKAIILQIDNKSQQVSASALRHSLQDRLSKGASGKNRDEINLKLRTSTAPPLKLVDLPGLDQRIVDESMIAEYAQHNDAILLVIVPASQASEISSSRALKIAKEYDPESTRTIGIIGKIDQAAENSKALAAVQALLSNQGPPKTTDIPWVAVIGQSVSIASAQSGSGENSLETAWRAESESLKSILTGAPQSKLGRIALVDTLASQIRSRMKLRLPSVLSGLQGKSQIVQDELARLGEQLVNSAEGTRAIALELCREFEDKFLLHLAGGEGSGWKVVASFEGNFPNRIKQLPLDRHFDLNNVKRVVLEADGYQPYLISPEKGLRSLIKIVLELAKDPARLCVDEVHRVLVDIVSASANATPGLGRYPPFKREVVAIASAALDGFKNEAKKMVVALVDMERAFVPPQHFIRLVQRRMERQRREEELKGRSSKKGQDAEQSLLSRATSPQPDGPTAGGSLKSMKDKPSPQDKETPEVSGLKTAGPEGEITAGYLMKKSAKTNGWSRRWFVLNEKTGKLGYTKKQEERNFRGTITLEECTIEEIPEDEVEKSKSSKDKKANGPDSKGPGLVFKITCKVPYKTVLKAHNALVLKAESVVDKNEWINKLQKVIQARGGQVGSVSMRQSLSEGSLDKMVRKPIDPEEELRWMSQEVRGYVEAVLNSLAANVPKAVVLCQVEKAKEDMLNQLYSSISAIGNERIESLIQEDQNVKRRRERYQKQSSLLSKLTRQLSIHDNRAAAASSYSDNSGTESpSPRASGGSSGDDWMNAFNSAANGPSDSLSKYGSGGHSRRYSDPAQNGDAASPGSGSNRRTTPNRLPPAPPPTGSAYRY |
| **AT4G38710** | MAAAVSSVWAKPGAWALEAEEHEAELKQQPSPTNQKSSAEDSSDFPSLAAAATTKTKKKKGQTISLAEFATYGTAKAKPAPQTERLTQAELVALPTGPRERSAEELDRSKLGGGFRSYGGGRYGDESSSSRWGSSRVSEDGERRGGGFNRDREPSRDSGPSRADEDDNWAAAKKPISGNGFERRERGSGGGFFESQSQSKADEVDSWVSTKPSEPRRFVSSNGGGGDRFEKRGSFESLSRNRDSQYGGGGGSESDTWGRRREESGAANGSPPPSGGSRPRLVLQPRTLPVAVVEVVKPEpSPVLVIVEKPKGANPFGNARPREEVLAEKGQDWKEIDEKLEAEKLKDIAAAMEKPNEKSTGKMGFGLGNGRKDEERIERSWRKSFSLHSYMEVDVLNTEHSEEDAQEEEPAVEGAKKEETEDKPAVEEAKKEETEGEQAVEEAKKEETGGEPAVEEAKKEETEDKI |
| **AT1G07110** | MGSGASKNTEEDDDGSNGGGGQLYVSLKMENSKVEGELTPHVYGSLPLIGSWDPSKALPMQRESALMSELSFVVPPDHETLDFKFLLKPKNRNTPCIVEEGENRLLTGGSLQGDARLALFRLEGDVIVEFRVFINADRVSPIDLATSWRAYRENLQPSTVRGIPDVSINPDPKSAECPLESLELDLAHYEVPAPAPSANSYLVYAADNAENPRSLSASGSFRNDSTPKAAQRNSEDSGVTVDGSPSAKEMTIVVPDSSNIYSAFGEAESKSVETLpSPFQQKDGQKGLFVDRGVGSPRLVKSLSASSFLIDTKQIKNSMPAAAGAVAAAAVADQMLGPKEDRHLAIVLVGLPARGKTFTAAKLTRYLRWLGHDTKHFNVGKYRRLKHGVNMSADFFRADNPEGVEARTEVAALAMEDMIAWMQEGGQVGIFDATNSTRVRRNMLMKMAEGKCKIIFLETLCNDERIIERNIRLKIQQSPDYSEEMDFEAGVRDFRDRLANYEKVYEPVEEGSYIKMIDMVSGNGGQIQVNNISGYLPGRIVFFLVNTHLTPRPILLTRHGESMDNVRGRIGGDSVISDSGKLYAKKLASFVEKRLKSEKAASIWTSTLQRTNLTASSIVGFPKVQWRALDEINAGVCDGMTYEEVKKNMPEEYESRKKDKLRYRYPRGESYLDVIQRLEPVIIELERQRAPVVVISHQAVLRALYAYFADRPLKEIPQIEMPLHTIIEIQMGVSGVQEKRYKLMD |
| **AT4G29810** | MKKGGFSNNLKLAIPVAGEQSITKFLRKGFGSLCRTQSGTFKDGDLRVNKDGVRIISQLEPEVLpSPIKPADDQLSLSDLDMVKVIGKGSSGVVQLVQHKWTGQFFALKVIQLNIDEAIRKAIAQELKINQSSQCPNLVTSYQSFYDNGAISLILEYMDGGSLADFLKSVKAIPDSYLSAIFRQVLQGLIYLHHDRHIIHRDLKPSNLLINHRGEVKITDFGVSTVMTNTAGLANTFVGTYNYMSPERIVGNKYGNKSDIWSLGLVVLECATGKFPYAPPNQEETWTSVFELMEAIVDQPPPALPSGNFSPELSSFISTCLQKDPNSRSSAKELMEHPFLNKYDYSGINLASYFTDAGSPLATLGNLSGTFSV |
| **AT1G59610** | MEAIDELSQLSDSMRQAASLLADEDPDETSSSRRPATSLNVVALGNVGAGKSAVLNSLIGHPVLPTGENGATRAPIIIDLSREESLSSKAIILQIDNKNQQVSASALRHSLQDRLSKGASGRGRDEIYLKLRTSTAPPLKLIDLPGLDQRIVDDSMIGEHAQHNDAILLVVVPASQASEISSSRALKIAKEYDPESTRTVGIISKIDQAAENPKSLAAVQALLSNQGPPKTTDIPWVALIGQSVSIASAQSGGSENSLETAWRAESESLKSILTGAPQSKLGRIALVDTLASQIRSRMKLRLPNILTGLQGKSQIVQDELARLGEQLVSSAEGTRAIALELCREFEDKFLLHLAGGEGSGWKVVASFEGNFPNRIKKLPLDRHFDLNNVKRIVLEADGYQPYLISPEKGLRSLIKTVLELAKDPARLCVDEVHRVLVDIVSASANATPGLGRYPPFKREVVAIASAALDGFKNEAKKMVVALVDMERAFVPPQHFIRLVQRRMERQRREEELKGRSSKKGQDAEQSLLNRATSPQPDGPSSTGGSLKSLRDKLMPQDKDKDKEKETPEVSGLKTAGPEGEITAGYLMKKSAKTNGWSRRWFVLNEKTGKLGYTKKQEERNFRGTVTLEECSIEEISDDEGEKSKSSKDKKSNGPDSKGPGLVFKITCRVPYKTVLKAHNALVLKAESMVDKNEWINKLQKVIQARGGQVGSASMRQSLSEGSLDKMVRKPVDPEEELRWMSQEVRGYVEAVLNSLAANVPKAVVLCQVEKSKEDMLNQLYSSISAIGNERIESLIQEDQNVKRRRDRYQKQSSLLSKLTRQLSIHDNRAAAASSWSDNSGTESpSPRTNGGSSGEDWMNAFNAAASGPDSLKRYGSGGHSRRYSDPAQNGEDSSGSGGSSRRTTPNRLPPAPPQSGSSYRY |
| **AT5G18230** | MGASRKLQGEIDRVLKKVQEGVDVFDSIWNKWNVYDTDNVNQKEKFEADLKKEIKKLQRYRDQIKTWIQSSEIKDKKVSASYEQSLVDARKLIEKEMERFKICEKETKTKAFSKEGLGQQPKTDPKEKAKSETRDWLNNVVSELESQIDSFEAELEGLSVKKGKTRPPRLTHLETSITRHKDHIIKLELILRLLDNDELSPEQVNDVKDFLDDYVERNQDDFDEFSDVDELYSTLPLDEVEGLEDLVTAGPLVKGTPLSMKSSLAASASQVRSISLPTHHQEKTEDTSLPDSSAEMVPKTPPPKNGAGLHSAPSTPAGGRPSLNVPAGNVSNTSVTLSTSIPTQTSIESMGSLSPVAAKEEDATTLPSRKPPSSVADTPLRGIGRVGIPNQPQPSQPPSPIPANGSRISATSAAEVAKRNIMGVESNVQPLTpSPLSKMVLPPTAKGNDGTASDSNPGDVAASIGRAFSPSIVSGSQWRPGSPFQSQNETVRGRTEIAPDQREKFLQRLQQVQQGHGNLLGIPSLSGGNEKQFSSQQQNPLLQQSSSISPHGSLGIGVQAPGFNVMSSASLQQQSNAMSQQLGQQPSVADVDHVRNDDQSQQNLPDDSASIAASKAIQSEDDSKVLFDTPSGMPSYMLDPVQVSSGPDFSPGQPIQPGQSSSSLGVIGRRSNSELGAIGDPSAVGPMHDQMHNLQMLEAAFYKRPQPSDSERPRPYSPRNPAITPQTFPQTQAPIINNPLLWERLGSDAYGTDTLFFAFYYQQNSYQQYLAAKELKKQSWRYHRKFNTWFQRHKEPKIATDEYEQGAYVYFDFQTPKDENQEGGWCQRIKNEFTFEYSYLEDELVV |
| **AT4G38550** | MDFFTSSKAKKDSKKSSGLFGKKTVSKSTPGSPAHPPGARpSPPPSYLSNKRAETEYDFPMSNEQRPYWKQPASERVPNSHPRPPVYGYGTPDHRRDHGRERMEAMSYEPETNAPSSPYHPAGNRTPERPRKSTEYRREHQDRMYEADTRSNASPFHPFRSPSPSPFHTPDRRRDHYDMYEPEANTMLQNSAPGSPFHPAGSRSPPPYRTPDRRSNYDKEQFEDLYEQDGDVTPRNSSPPpSPFHPAAYKTSDQRSNHGKEQIEDFYEQDDDVTPRNSSPPSPLHPAASHSPPPPQPYRTPDHRRSHQDNEDFEAMYELDGDLIHQKSAPPSPVHGPYYSSSDDDNHSTYLYPEIRSPLRSRIVSENSTPVHHNYQIVAAETYEQDKQFEPPELPDESQSFTMQEITKMRGLKNYESGKEESQSMISEAYVSVANYRVRQSVSETLQAIIDKHGDIAASSKLQAMATRSYYLESLAAVVMELKKTVLRDLTKTRVAEIAAVVKDMESVKINVSWLKTAVTELAEAVEYFGQYDTAKVEKEVCERDLTAKKGEMEEMTAELVKREKEIKECREKVTVVAGRLGQLEMKGSKLNKNLDLFQSKVHKFQGEAVLLHL |
| **AT1G48620** | MDPSLSATNDPHHPPPPQFTSFPPFTNTNPFASPNHPFFTGPTAVAPPNNIHLYQAAPPQQPQTSPVPPHPSISHPPYSDMICTAIAALNEPDGSSKQAISRYIERIYTGIPTAHGALLTHHLKTLKTSGILVMVKKSYKLASTPPPPPPTSVAPSLEPPRSDFIVNENQPLPDPVLASSTPQTIKRGRGRPPKAKPDVVQPQPLTNGKLTWEQSELPVSRPEEIQIQPPQLPLQPQQPVKRPPGRPRKDGTpSPTVKPAASVSGGVETVKRRGRPPSGRAAGRERKPIVVSAPASVFPYVANGGVRRRGRPKRVDAGGASSVAPPPPPPTNVESGGEEVAVKKRGRGRPPKIGGVIRKPMKPMRSFARTGKPVGRPRKNAVSVGASGRQDGDYGELKKKFELFQARAKDIVIVLKSEIGGSGNQAVVQAIQDLEGIAETTNEPKHMEEVQLPDEEHLETEPEAEGQGQTEAEAMQEALF |
| **AT4G15545** | MSEIEEEEEEGSASAITGSRSFDLPDELLQVLPSDPFEQLDVARKITSIALSTRVSALESESSDLRELLAEKEKEFEELQSHVESLEASLSDAFHKLSLADGEKENLIRENASLSNTVKRLQRDVSKLEGFRKTLMMSLQDDDQNAGTTQIIAKPTPNDDDTPFQPSRHSSIQSQQASEAIEPAATDNENDAPKPSLSASLPLVSQTTpTPRLTPPGSPPILSASGTPKTTSRPISPRRHSVSFATTRGMFDDTRSSISISEPGSQTARTRVDGKEFFRQVRSRLSYEQFGAFLGNVKDLNAHKQTREETLRKAEEIFGGDNRDLYVIFEGLITRNAH |
| **AT1G27100** | MELFTKGNNVKLRSHLDKFLVADDDQETIRQSRKGDARRAVWTVEPVVDKPNLIRLKSSHGTYLTASNKPLLLGMTGEKVTQTASFNKLMDWQTQWEPERDGFQVKLKSWCGKWMRANGGTPPWRNSVTHDEPHTSKTKNWLIWDVITIDGSDLENMSDGDESSVSSPVGSEFGSEPGSPVSARSTKSSISRFASLGLSTSPRWSSKPKSTASSFNQKETPVSVSAMEFFQKAKAIRMRNSHNKYLTADDDEETVTQNRNGSTKNARWTVEPVRDSFHVIRLKSCYGKYLTASNERFLLGATGKKVIQLKLSRVDSSVEWEPLREGSKIKLRTRSGNYLRGNGGLPPWRNSVTHDVPHLSATQDSISWDVDVVEILTDSEFKTESAKAPPPKTTSPPPHRRPTSSPLSAEpSPRTSSSLSDRSDSDSVESPPKSDGRTIYYHVADEEGHVEDETTVGYAFTFKGNSVAELTQTLREETCMEDAVVCTRSPLNGKLFPLRLQLPPNNGTLHVILLPSSASL |
| **AT5G12850** | MCGLAKKLDIEDTLTSLSDQENESLAKPMNDAAEWEHSFSALLEFAADNDVEGFRRQLSDVSCINQMGLWYRRQRFVRRMVLEQRTPLMVASLYGSLDVVKFILSFPEAELNLSCGPDKSTALHCAASGASVNSLDVVKLLLSVGADPNIPDAHGNRPVDVLVVSPHAPGLRTILEEILKKDEIISEDLHASSSSLGSSFRSLSSSPDNGSSLLSLDSVSSPTKPHGTDVTFASEKKEYPIDPSLPDIKSGIYSTDEFRMFSFKIRPCSRAYSHDWTECPFAHPGENARRRDPRKFHYTCVPCPDFKKGSCKQGDMCEYAHGVFECWLHPAQYRTRLCKDGMGCNRRVCFFAHANEELRPLYPSTGSGLPSPRASSAVSASTMDMASVLNMLPGSPSAAQHSFTPPISPSGNGSMPHSSMGWPQQNIPALNLPGSNIQLSRLRSSLNARDIPSEQLSMLHEFEMQRQLAGDMHSPRFMNHSARPKTLNPSNLEELFSAEVApSPRFSDQLAVSSVLSPSHKSALLNQLQNNKQSMLSPIKTNLMSSPKNVEQHSLLQQASSPRGGEPISPMNARMKQQLHSRSLSSRDFGSSLPRDLMPTDSGSPLSPWSSWDQTHGSKVDWSVQSDELGRLRKSHSLANNPNREADVSWAQQMLKDSSSPRNGNRVVNMNGARPLTQGGSSVNPHNSDTRESDILDAWLEQLHLDR |


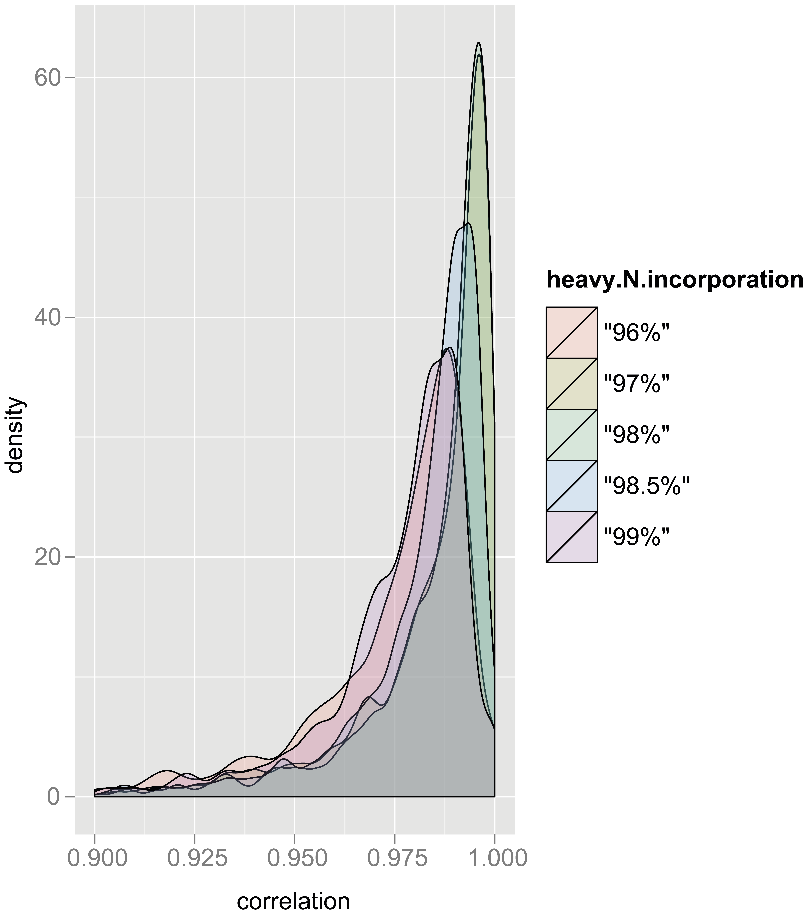


Figure S1: Density plots of all correlations between expected and measured peptide isotope envelopes in shotgun proteomics analyses considering 96% to 99% stable heavy nitrogen isotope (^15^N) incorporation into proteins. The density plot that most approximates perfect correlation can be sued as an estimate of heavy isotope incorporation.


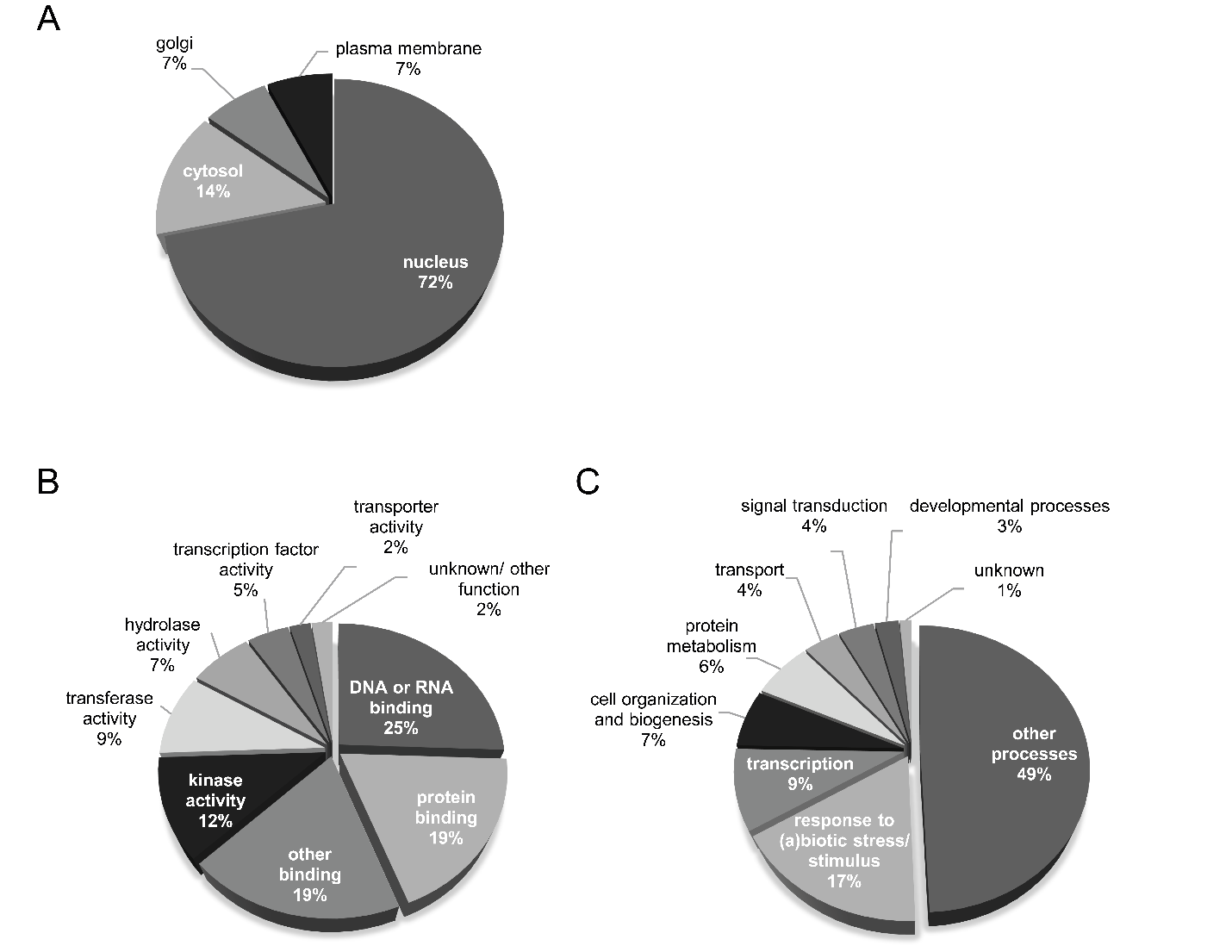


Figure S2:

GO annotation analyses of all direct targets of MKK7-MPK3/6. (A) Cellular localization (B) Molecular function (C) Cellular process.


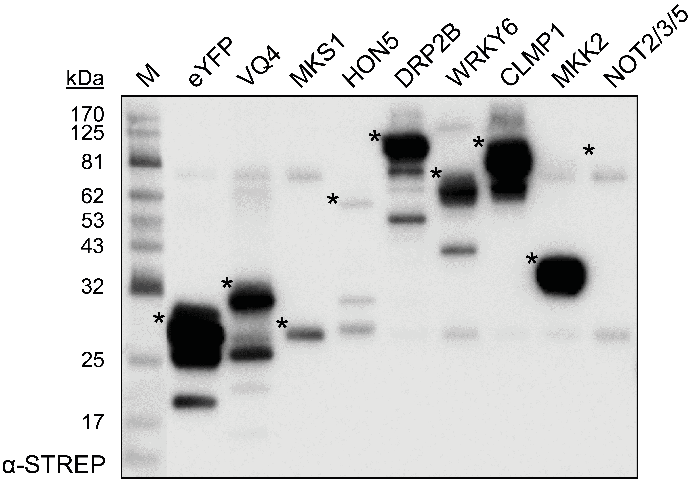


Figure S3:

Purification of all StrepII-tagged putative MPK substrates used in this study by affinity chromatography and analysed by immunodetection using a specific α-STREP antibody.


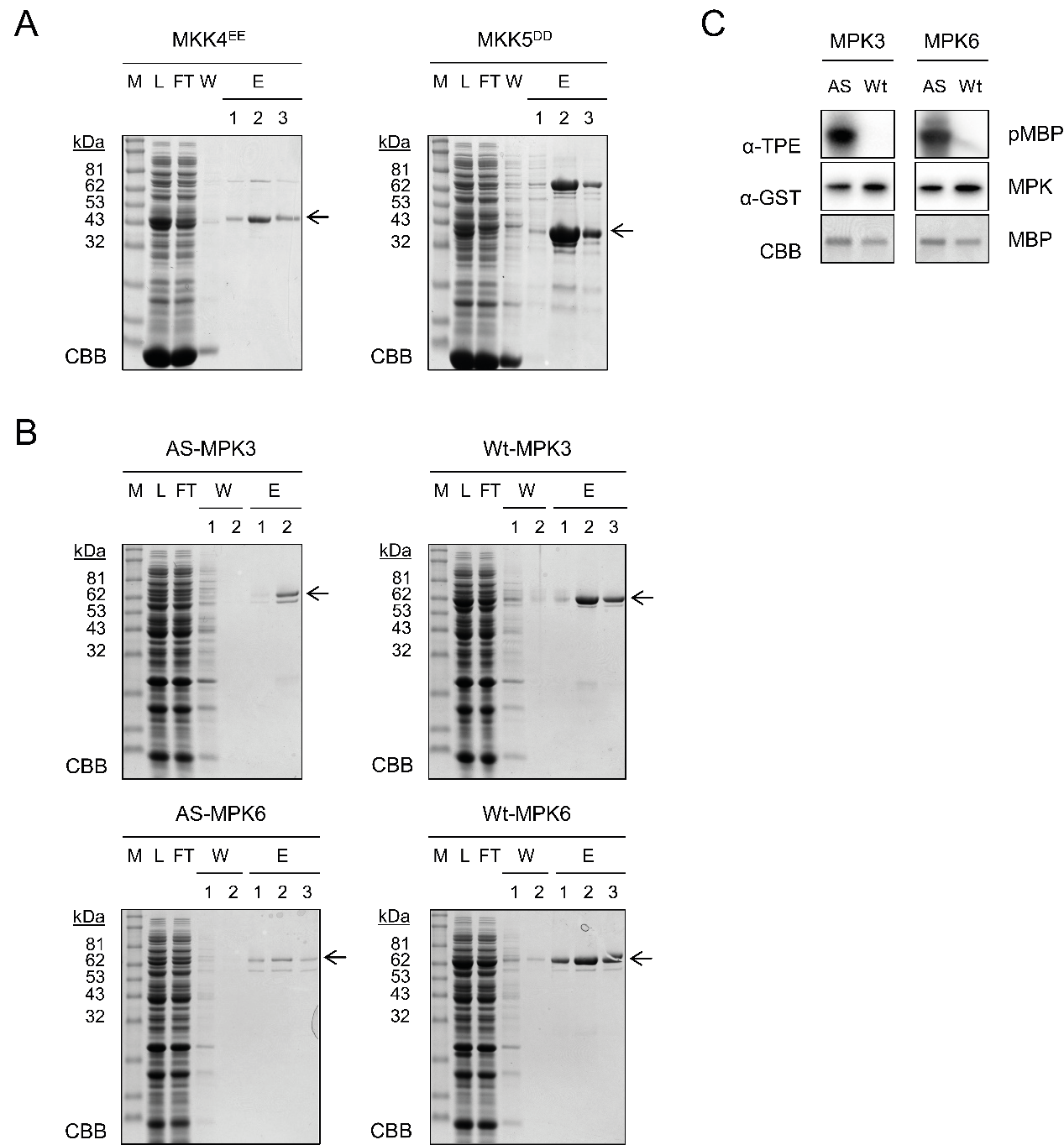


Figure S4:

(A) Exemplary purification of GST-tagged MPK3/6 and His6-tagged MKK4/5 used in this study.

(B) Exemplary activity assay of purified Wt- and AS-MPK3/6 using MBP as substrate.
